# Accuracy of Blood Loss Estimation and Identification of Factors Contributing to Early Postpartum Hemorrhage Following Vaginal Delivery

**DOI:** 10.3390/jcm15083000

**Published:** 2026-04-15

**Authors:** Gabriela Afrykańska, Maja Kłopecka, Hanna Maciocha, Julia Wyszyńska, Zofia Włodarczyk, Szymon Paruszewski, Aleksandra Maria Śliwka, Artur Arkadiusz Ludwin, Paweł Jan Stanirowski

**Affiliations:** 1st Department of Obstetrics and Gynecology, Medical University of Warsaw, 02-015 Warsaw, Poland

**Keywords:** postpartum hemorrhage, vaginal delivery, blood loss estimation, risk factors

## Abstract

**Objective:** The study aimed to assess the accuracy of two distinct methods for estimating blood loss (EBL) and to identify potential factors contributing to early-onset postpartum hemorrhage (PPH) following a vaginal delivery (VD). **Methods:** Women in singleton pregnancies undergoing spontaneous/induced VD were recruited for this prospective observational cohort study. Methods of EBL included: (1) visual assessment by an attending obstetrician (sEBL) and (2) implementation of a mathematical formula (fEBL). Early PPH was defined as a cumulative blood loss exceeding 500 mL within the first 24 h after delivery as reflected by clinical assessment. **Results:** During the study period, 485 women delivered vaginally, and early PPH was diagnosed in 29 cases (5.97%). Among patients with PPH, a significant increase in the duration of the 2nd (61 min. vs. 33.5 min., *p* < 0.05) and 3rd (13 min. vs. 7 min., *p* < 0.001) stages of labor, as well as in the application of a dinoprostone insert (31% vs. 10.5%, *p* < 0.01) was noted. Additionally, in the same cohort, uterine atony (41.4% vs. 1.5%, *p* < 0.001), 3rd/4th degree perineal rupture (6.9% vs. 0%, *p* < 0.01), fetal macrosomia (17.2% vs. 4.8%, *p* < 0.05) and stillbirth (6.9% vs. 0.2%, *p* < 0.05) occurred significantly more frequently. In both groups visual estimation of blood loss was significantly lower compared to fEBL: (PPH sEBL: 800 mL vs. fEBL 1439.6 mL, *p* < 0.001; control sEBL: 250 mL vs. fEBL 621.8 mL, *p* < 0.001). In the multivariate analysis, factors such as third stage of delivery time ≥ 30 min. (OR 11.6; 95% CI: 4.18–32.33), FBW ≥ 4000 g (OR 6.37; 95% CI: 1.54–26.3), and dinoprostone insert application (OR 4.33; 95%CI: 1.63–11.48) were selected as independent predictors of the PPH. **Conclusions:** Compared to mathematical formula, visual estimation of blood loss by an attending obstetrician is significantly decreased. Prolonged third stage of delivery, fetal macrosomia, and application of a dinoprostone insert are the strongest contributors to early PPH following a VD.

## 1. Introduction

Postpartum hemorrhage (PPH) constitutes the major cause of maternal mortality and morbidity worldwide, accounting for 8% to 30% of maternal deaths [1,2,3]. According to widely accepted World Health Organization (WHO) standards, early PPH is defined as a blood loss exceeding 500 mL within 24 h of childbirth [4], whereas the American College of Obstetricians and Gynecologists (ACOG) adopts a higher threshold of ≥1000 mL within the same timeframe [5]. The main rationale for the latter criterion is to provide a uniform definition of hemorrhage irrespective of the route of delivery, and thus to reduce potential overclassification. From the clinical perspective, such discrepancies across diagnostic criteria lead to considerable variability in the recorded prevalence of PPH, while simultaneously impeding the execution of direct cross-study evaluations [6].

Despite increasing Cesarean section (CS) rates, vaginal delivery (VD) continues to represent the optimal and safest mode of childbirth for most gravid individuals. Nonetheless, even in the natural course of labor, complications may occur, with PPH affecting approximately 1.2–12.6% of parturients [7,8,9]. To effectively diminish the incidence of considerable blood loss, numerous preventive modalities, including pharmacological agents like uterotonics and antifibrinolytics, in addition to mechanical interventions such as balloon tamponades, have been incrementally incorporated into clinical practice [10,11]. Despite the undoubted efficacy of the above-mentioned treatment, the urgency and unpredictable course, as well as high morbidity and mortality rates, make the effective management of PPH a persistent global challenge, underscoring the need for improved protocols and more accurate identification of risk factors.

Effective strategies for addressing PPH are critically dependent on the early and accurate determination of estimated blood loss (EBL). Visual estimation conducted by an attending obstetrician (sEBL) constitutes the most frequently employed technique for assessing EBL, yet this approach is intrinsically subjective and susceptible to reporting minor volumes as higher and significant volumes as lower than actual [12]. Due to the identified inaccuracies, novel methodologies were adopted in clinical settings; these included mathematical formulas (fEBL) which utilize maternal pre- and post-delivery laboratory parameters, gravimetric methods employing blood-soaked dressings, and collection bags for direct volume measurement [13,14]. While the latter two methods offer higher precision, they require additional training and resource allocation, are time-consuming and are prone to inaccuracies due to contamination with amniotic fluid or urine [15]. The remaining approach—fEBL—although based on theoretical assumptions, exhibits a higher degree of standardization, consequently rendering it more objective when compared to sEBL [16,17]. Moreover, the implementation of the algorithm necessitates neither supplementary expenditures nor expertise, making it suitable for low-resource settings.

Apart from accurate estimation of blood loss, early identification of contributing factors plays a pivotal role in PPH management. Excessive postpartum bleeding is frequently attributed to a specific cluster of pathologies, collectively designated as the 4 “T’s” group, which encompasses inadequate uterine contractility, birth canal lacerations, placental retention, and coagulation abnormalities [18]. In accordance, existing literature identifies factors such as multiple pregnancy, prolonged second stage of labor, previous history of PPH, and maternal anemia as bearing the highest propensity for inducing PPH [19,20,21]. From a clinical standpoint, understanding the principal factors contributing to obstetric hemorrhage facilitates the timely stratification of women at high risk and implementation of anticipatory birth planning.

The objective of the presented study was twofold: firstly, to evaluate independent factors contributing to early-onset PPH, and secondly, to compare the accuracy of different methods of blood loss estimation following a VD within a tertiary care center. The fulfillment of these objectives is intended to enhance obstetric care by enabling more prompt and accurate identification of PPH, thereby facilitating clinical decision-making and reducing the rate of adverse perinatal outcomes.

## 2. Materials and Methods

### 2.1. Study Design and Population

A prospective observational cohort study was conducted between 1 January 2023 and 28 February 2024 within the 1st Department of Obstetrics and Gynecology, a tertiary referral institution at the Medical University of Warsaw performing approximately 1300 deliveries per annum. The research protocol received approval from the Local Ethics Committee (reference no. KB/36/2022, dated 14 March 2022), and written informed consent was acquired from all individuals participating in the study. The analysis was restricted to women with singleton pregnancies who delivered vaginally, and for whom both visual and calculated methods for EBL were accessible. Patients qualified for elective or emergency CS, those with multifetal gestations, and subjects exhibiting known coagulopathies were excluded from the study.

In accordance with the recommendations of the Polish Society of Gynecologists and Obstetricians, preventive measures against PPH were implemented in all laboring women [22]. Namely, in patients without known risk factors for PPH a prophylactic dose of oxytocin was administered after the delivery of the fetus, whereas in women presenting established risk factors for uterine atony (e.g., uterine myomas, polyhydramnios, fetal macrosomia, history of PPH or uterine atony), carbetocin was provided.

In each participant two methods of EBL were evaluated and compared: (1) visual estimation of blood loss by attending obstetrician (sEBL) and (2) the application of a mathematical formula for EBL calculation (fEBL). To perform the latter, concentrations of erythrocytes, hemoglobin (Hgb), and hematocrit (Hct) were measured in each participant prior to the VD and reevaluated 24 h after the labor. A formula accessible on the website was employed for blood loss estimation [14]. Metric units representing maternal weight and height were converted into imperial units: 1 pound = 2.2 × 1 kg and 1 inch = 39.37 × 1 m. The equations used for the fEBL calculation are given below:

1. Calculated pregnancy blood volume = 0.75 × ([maternal height (inches) × 50] + [maternal weight (pounds) × 25]);
2. Percentage of blood volume lost = (pre-delivery Hct − post-delivery Hct)/pre-delivery Hct;
3. fEBL = Calculated pregnancy blood volume x percentage of blood volume lost.

### 2.2. Study Outcome Definitions

Considering that only VD were included, early PPH was defined as a cumulative blood loss exceeding 500 mL within the first 24 h after delivery as reflected by clinical assessment (sEBL), and according to WHO standards [4,23]. As part of an exploratory analysis, obstetric outcomes among patients who, according to a mathematical formula, lost more than 1000 mL of blood, yet were not clinically diagnosed with hemorrhage by an obstetrician (potentially undiagnosed hemorrhage), were evaluated. Pre-gestational diabetes mellitus (PGDM) encompassed cases of both type 1 and type 2 diabetes mellitus diagnosed prior to pregnancy. Dinoprostone insert was applied to women with Bishop score ≤ 6 points without prior uterine surgery [24]. In women with favorable cervix for labor induction (Bishop > 6 points), either a Foley catheter, amniocentesis or oxytocin administration was introduced [20]. The total time of delivery was derived by summing the periods corresponding to the first, second, and third stages of labor. Consistent with national guidelines, the permissible upper limit for the second stage of labor was determined to be 120 min, and increasing to 180 min for cases involving epidural anesthesia [25]. The diagnosis of retained placenta or retained products of conception (RPOC) was established when either the entire or part of the placenta/membranes failed to undergo spontaneous expulsion within 30 min of the third stage of labor. Fetuses were classified as growth-restricted (FGR) according to the criteria proposed by the Delphi consensus [26]. Fetuses with estimated birth weight between the 3rd and 10th percentile for gestational age who did not meet the Delphi criteria were classified as small-for-gestational-age (SGA). A diagnosis of fetal macrosomia was established when the fetal birthweight (FBW) exceeded 4000 g, irrespective of gestational age. Stillbirth was defined as the death of the fetus after 22 + 0/7 gestational weeks. Finally, the designation “night shift” was applied to VD that manifested during the nocturnal period, specifically from 22:00 to 07:00 on the following day.

### 2.3. Statistical Analysis

All calculations were performed using R software (R Core Team, Vienna, Austria), version 4.3.2. Continuous variables were compared using Student’s *t*-test or the Mann–Whitney U test following distribution analysis. To evaluate disparities in qualitative data, either the chi-square test or Fisher’s exact test was employed. The Wilcoxon rank sum test with Bonferroni correction was used to compare paired EBL values in each study group. Continuous data were expressed as the mean, standard deviation (SD), and range or as the median and interquartile range (IQR). In the case of categorical data, frequency distributions were presented as percentages (%).

Spearman’s rank correlation coefficient (rho) was used to examine monotonic relationships between selected continuous variables and methods of EBL. Results were visualized using heatmaps, where correlation strength was represented by the size and color of the points.

In order to assess the associations between selected clinical parameters and PPH occurrence, univariate and multivariate logistic regression models were employed. In univariate models, each variable was assessed separately, while in multivariate analysis, multiple variables were entered simultaneously to control for potential confounding. Candidate variables for the multivariate logistic regression model included both ante- and intrapartum factors which were chosen based on clinical relevance and their potential association with PPH. The initial model included prespecified candidate predictors, and backward stepwise selection based on the Akaike Information Criterion (AIC) was applied to obtain the final model. Multicollinearity was assessed using generalized variance inflation factors (GVIF). Lastly, for the evaluation of model’s performance and discriminative capacity the analysis of receiver operating characteristic (ROC) curve was conducted, and area under the curve (AUC) was calculated.

The results were considered statistically significant if the *p*-value was <0.05.

## 3. Results

During the study period, there were 1341 deliveries in the Department, including 507 VD (37.8%). The additional exclusion of 114 multiple pregnancies led to a dataset of 1227 singleton deliveries, among which 501 (40.8%) were VD. This cohort was further restricted as two individuals declined participation, four medical records lacked documentation of sEBL, and ten cases failed to have pre-delivery blood samples collected, rendering the computation of fEBL impossible. Following the completion of accessible data, 29 cases of early PPH (29/485, 5.97%) were diagnosed and compared to 456 controls.

The study participant characteristics are presented in Table 1, Table 2 and Table 3 and Supplementary Table S1. Gravidity and parity were found to be substantially lower among women with a PPH diagnosis, relative to the control population (*p* < 0.05). No statistically significant differences were observed between the two cohorts concerning gestational age at delivery (PPH 40 weeks [39–40] vs. control 39 weeks [39–40], *p* = 0.26), nor in maternal parameters including pre-pregnancy body mass index (BMI) (PPH 22.3 kg/m^2^ [20.7–24.6] vs. control 22.3 kg/m^2^ [20.4–25], *p* = 0.88) and gestational weight gain (GWG) (PPH 14 kg [10–18] vs. control 13 kg [10–17], *p* = 0.06). In addition, maternal and fetal adverse outcomes, including preterm delivery, preterm rupture of membranes, preeclampsia, gestational diabetes mellitus, PGDM, pregnancy-induced hypertension, chronic hypertension, FGR, and SGA did not show statistically significant variations between the PPH and control groups (Table 1, Table 2 and Table 3). While the proportion of induced VD appeared greater within the cohort of women experiencing hemorrhage, observed discrepancy lacked statistical significance (PPH 48.3% vs. control 35.3%, *p* = 0.23) (Table 2). Dinoprostone insert constituted the predominant method for labor preinduction within the PPH cohort, demonstrating a significantly higher rate of application compared to the control group (PPH 64.3% vs. control 29.2%, *p* < 0.05) (Table 2).

Regarding labor progression, no statistically significant differences were identified in the duration of the first stage of delivery (PPH 270 min. [170–375] vs. control 270 min. [173.7–420], *p* = 0.91) (Table 2). In contrast, women presenting with postpartum hemorrhage exhibited a markedly extended duration for both the 2nd (PPH 61 min. [25–99] vs. control 33.5 min. [16–70], *p* < 0.05) and 3rd (PPH 13 min. [8–30] vs. control 7 min. [5–10], *p* < 0.001) stages of labor. A prolonged 2nd (≥180 min) and 3rd (≥30 min) stage was observed in 10.3% and 27.6% of PPH cases, as compared to 1.8% and 3.7% in the control group, respectively, (*p* < 0.05). Comparison between the PPH and non-PPH groups demonstrated no significant differences in the total duration of the labor, percentage of episiotomies (PPH 37.9% vs. control 33.1%, *p* = 0.74) as well as in the proportion of night shift deliveries (PPH 44.8% vs. control 31.8%, *p* = 0.21), vacuum deliveries (PPH 13.8% vs. control 7.7%, *p* = 0.28), or VD with epidural anesthesia (PPH 86.2% vs. control 71.1%, *p* = 0.09)

Women diagnosed with early-onset PPH exhibited a significantly higher incidence of VD complications, including 3/4 degree perineal ruptures (PPH 6.9% vs. control 0, *p* < 0.01), uterine atony (PPH 41.4% vs. control 1.5%, *p* < 0.001), retained placenta (PPH 27.6% vs. control 2.2%, *p* < 0.001), and RPOC (PPH 62.1% vs. control 7.5%, *p* < 0.001), compared to the control group (Table 2). Consequently, in the former group the proportion of patients necessitating the administration of uterotonic agents (carbetocin, methergine, misoprostol; *p* < 0.001), antifibrinolytics (tranexamic acid; *p* < 0.001), additional invasive procedures (curettage, Crede maneuver, manual removal of the placenta; *p* < 0.001), and blood transfusion (PPH 20.7% vs. control 0.9%, *p* < 0.001) was significantly higher, in comparison to the control cohort.

The analysis of maternal laboratory data indicated no significant intergroup variation in pre-delivery erythrocyte count, Hgb concentration, or Hct values (Supplementary Table S1). In contrast, women experiencing PPH displayed a substantial post-delivery reduction in the above-mentioned variables (*p* < 0.001). Regarding neonatal outcomes, a statistically significant increase in the proportion of macrosomic fetuses ≥ 4000 g was observed among patients diagnosed with PPH (17.2% vs. 4.8%, *p* < 0.05) (Table 3). Additionally, in the same subgroup stillbirth occurred more frequently (6.9% vs. 0.2%, *p* < 0.05).

Upon completion of the analysis, a substantial group of participants (n = 86) lost more than 1000 mL of blood according to a calculated formula, yet were not clinically diagnosed with hemorrhage by the obstetrician. Those patients were classified as having potentially undiagnosed hemorrhage (sEBL < 500 mL, fEBL ≥ 1000 mL) and compared to patients without PPH. In the former group a significant increase in the GWG (14 kg [11–17] vs. 12 kg [10–16]; *p* < 0.05), duration of the 2nd stage of delivery (55 min. [25.5–95.5] vs. 30 min. [15.2–63]; *p* < 0.001), total time of delivery (375 min. [251.2–590] vs. 313.5 min. [201.5–460]; *p* < 0.05), and rate of episiotomy (59.3% vs. 27%; *p* < 0.001) was noted.

A comparative assessment of EBL methodologies demonstrated a statistically significant reduction in sEBL measurements compared to fEBL in both the PPH and control cohorts (PPH sEBL: 800 mL [600–1000] vs. fEBL 1439.6 mL [1114.3–1673.6], *p* < 0.001; control sEBL: 250 mL [200–300] vs. fEBL 621.8 mL [330.1–920], *p* < 0.001) (Table 4). In concordance, fEBL values were significantly higher than sEBL among women with potentially undiagnosed hemorrhage (fEBL 1199.6 mL [1100.2–1380.9] vs. sEBL 300 mL [262.5–350]; *p* < 0.001) as well as without PPH (fEBL 521.5 mL [273.8–735.6] vs. sEBL 250 mL [200–300], *p* < 0.001).

Correlation analysis performed in the total study population revealed the presence of positive associations between both methods of EBL and GWG (fEBL rho = 0.127; *p* < 0.01; sEBL rho = 0.091; *p* < 0.05), the duration of the 1st (fEBL rho = 0.143; *p* < 0.01; sEBL rho = 0.186; *p* < 0.001), 2nd (fEBL rho = 0.257; *p* < 0.001; sEBL rho = 0.278; *p* < 0.001) and 3rd (fEBL rho = 0.125; *p* < 0.01; sEBL rho = 0.193; *p* < 0.001) stages of delivery, total time of delivery (fEBL rho = 0.191; *p* < 0.001; sEBL rho = 0.233; *p* < 0.001), FBW (fEBL rho = 0.115; *p* < 0.05; sEBL rho = 0.115; *p* < 0.05), as well as differences in levels of erythrocytes (fEBL rho = 0.969; *p* < 0.001; sEBL rho = 0.395; *p* < 0.001), Hgb (fEBL rho = 0.954; *p* < 0.001; sEBL rho = 0.408; *p* < 0.001) and Hct (fEBL rho = 0.983; *p* < 0.001; sEBL rho = 0.398; *p* < 0.001) (Supplementary Figure S1A). Parity was the only variable negatively associated with both fEBL and sEBL methods (fEBL rho = −0.305; *p* < 0.001; sEBL rho = −0.312; *p* < 0.001). A subsequent subgroup analysis confirmed the presence of analogous correlative patterns among women without PPH (Supplementary Figure S1B). Conversely, within the cohort of individuals diagnosed with hemorrhage, the sole statistically significant correlations observed were confined to fEBL and the duration of the 3rd stage of delivery (fEBL rho = 0.475; *p* < 0.01), and differences in concentrations of erythrocytes (fEBL rho = 0.936; *p* < 0.001), Hgb (fEBL rho = 0.950; *p* < 0.001), and Hct (fEBL rho = 0.956; *p* < 0.001) (Supplementary Figure S1C).

In the univariate logistic regression analysis factors such as dinoprostone insert (OR 3.83; 95% CI: 1.65–8.87; *p* < 0.01), prolonged 2nd stage of delivery ≥ 180 min. (OR 6.6; 95% CI: 1.64–26.6; *p* < 0.01), prolonged 3rd stage of delivery ≥ 30 min. (OR 9.84; 95% CI: 3.81–25.4; *p* < 0.001), difference in erythrocytes concentration ≥ 0.7 mln/dL (OR 11.9; 95% CI: 4.73–30; *p* < 0.001), difference in Hgb concentration ≥ 2 g/dL (OR 12.9; 95% CI: 4.8–34.4; *p* < 0.001), difference in Hct ≥ 7% (OR 12.3; 95% CI: 5.3–28.8; *p* < 0.001), fetal macrosomia (OR 3.07; 95% CI: 0.86–11; *p* < 0.05), and stillbirth (OR 4.93; 95% CI: 0.3–38.3; *p* < 0.01) were associated with increased risk of PPH (Table 5).

A multivariate model selected five independent predictors of PPH when accounting for potential confounding (Table 6). Third stage of delivery time ≥ 30 min. (OR 11.62; 95% CI: 4.18–32.33), FBW ≥ 4000 g (OR 6.37; 95% CI: 1.54–26.3), and dinoprostone insert application (OR 4.33; 95%CI: 1.63–11.48) were the strongest contributors to hemorrhage following a VD. No relevant multicollinearity was identified, as the adjusted GVIF values were low. Due to the limited number of PPH cases, interaction terms were not tested in order to mitigate the risk of excessive model complexity and overfitting.

## 4. Discussion

This prospective observational cohort study aimed at comparing and evaluating the accuracy of two commonly used methods for assessing blood loss, as well as identifying factors contributing to early postpartum hemorrhage following vaginal delivery. The prevalence of early PPH during the study period amounted to 5.97%, which is similar to previous observations [21,27]. The conducted analysis demonstrated that visual evaluation of blood loss by the obstetrician and calculated assessment by the mathematical formula differ significantly, with the former method likely underestimating and fEBL overestimating lost blood volume. In addition, the multivariate analysis identified prolonged third stage of delivery, fetal macrosomia, and dinoprostone insert application as the independent determinants of early PPH.

Investigations have consistently identified sEBL as a subjective evaluation technique, demonstrating its propensity to underestimate the quantity of blood lost by approximately 20–50% [28,29,30,31]. Notably, the research by Legendre et al. identified a bimodal pattern of error in which medical practitioners tended to overestimate low blood volumes by up to 11.1% while underestimating higher volumes [12]. This lack of precision is further confirmed by recent comparative data, which shows that while objective methods identified PPH in 26.6% of deliveries, visual estimation captured only 5.1% of those cases [32]. In accordance with those findings in our previous study on the population of women who underwent CS, sEBL yielded substantially reduced estimations when juxtaposed with fEBL and the visual assessment of surgical dressings [33].

One of the key findings of the current investigation is the large fraction of potentially undiagnosed hemorrhage cases, characterized by fEBL greater than 1000 mL that remained unrecognized by the obstetrician. It is conceivable that the observed pattern is attributable to our institution’s policy, according to which the majority of VD are attended by residents at the beginning of professional medical training. As a result, visual estimation of the blood loss and most episiorrhaphy procedures are performed by individuals with insufficient clinical expertise. The observation that episiotomy remained a significant discriminatory factor between potentially undiagnosed and non-hemorrhage cases further supports this hypothesis as younger doctors need more time for perineal repair, which results in higher blood loss that is deliberately or unintentionally underestimated. Contrarily to our assumptions, previous studies have demonstrated that the error in EBL is predominantly dependent on the actual volume lost instead of experience, with medical students, residents and consultants exhibiting similar degrees of inaccuracy when relying solely on visual assessment [29,30,34]. Importantly, these errors can be significantly reduced through even minimal educational interventions, which underscores the need for consistent and standardized training. Collectively, the above-mentioned findings demonstrate that depending solely on sEBL may lead to overlooking of PPH cases, thus emphasizing the necessity of incorporating additional methods of assessment.

In contrast to conventional visual estimation, fEBLis defined as an algorithmically computed value that ascertains the difference in maternal hematocrit concentrations measured both ante- and postpartum. This approach facilitates standardization and objectivity in evaluation, thus leading to improved accuracy [35]. Nonetheless, an important limitation of this methodology lies in the delayed acquisition of laboratory diagnostic data and its inherently retrospective character, which precludes the prompt commencement of therapeutic interventions. In addition, theoretical assumptions leading to development of formula do not account for individual variations, including hemodynamic responses to hemorrhage, hydration status or shifts in postpartum plasma volume. Finally, some recently published studies provide evidence that mathematically estimated blood loss significantly exceeds the actual one, indicating a systemic overestimation [16,36]. Our investigation provided partial confirmation of this observation, with the median fEBL value of 621.8 mL within the cohort of women categorized by the protocol as not experiencing PPH. Likewise, in the study by Stafford et al., the authors demonstrated that fEBL was 2-fold higher than sEBL for operative vaginal delivery and 3-fold higher following VD complicated by laceration [16].

The existing body of research indicates that uterine atony constitutes the most critical cause of PPH, responsible for roughly 40–75% of cases [19,37,38,39]. In our population over 40% of women presenting with excessive postpartum bleeding experienced impaired contractility of the uterus. This relatively low prevalence of uterine atony may result from the recent implementation of Polish national guidelines advocating for routine carbetocin prophylaxis among high-risk individuals [22]. The efficacy of this approach is well-documented, as evidence from a recent meta-analysis that demonstrated a significantly lower incidence of severe PPH following carbetocin use compared to placebo or no intervention (RR 0.52; 95%CI: 0.37–0.73) [40].

The multivariate regression model indicated that a prolonged 3rd stage of labor is the most significant risk factor for PPH following a VD. Consistent with our findings, a recent analysis demonstrated that a 3rd stage of labor lasting ≥15 min was associated with a 5.5-fold higher risk of PPH [41], whereas a duration exceeding 30 min was associated with a more than sixfold increased risk of massive postpartum bleeding [42]. In addition, Voillequin et al. confirmed this association, and reported an increased risk of PPH in both nulliparous and multiparous women (aOR 1.80, 95% CI:1.37–2.38, and 2.10, 95% CI:1.56–2.83, respectively) [43]. The observed increase in the risk of PPH highlights the importance of the duration of the final stage of delivery as a clinically relevant parameter. Prolonged expulsion of the products of conception may reflect underlying abnormalities in placental separation or uterine contractility, which are known to impair hemostasis and contribute to excessive postpartum bleeding [44,45]. ACOG recognizes prolonged labor, labor induction, and prolonged oxytocin use as risk factors for uterine atony, suggesting that a prolonged 3rd stage of VD may be part of a broader pattern of dysfunctional labor predisposing to PPH [5].

Fetal macrosomia ≥ 4000 g represents another significant risk factor for early postpartum hemorrhage. In the present study, a higher median fetal birthweight was observed among women experiencing excessive bleeding, with 17.2% of neonates weighing above 4000 g. Our results are consistent with those of other authors, who also reported fetal overgrowth as a significant risk factor for PPH [39,46,47]. The proposed pathophysiological mechanism behind the process involves excessive uterine distention and prolonged labor. Impaired myometrial contractility resulting from uterine overdistention, combined with more frequent labor obstruction, increases the risk of both uterine atony and birth canal lacerations. 

Finally, the administration of a dinoprostone insert for labor preinduction was identified as a significant prognostic factor for early-onset PPH. Uterine hyperstimulation is a recognized adverse effect of dinoprostone, manifesting as excessive myometrial activity during labor, which can induce myometrial fatigue, and thus impair effective uterine contraction and postpartum involution [48,49]. Contrary to women diagnosed with hemorrhage, in the majority of uncomplicated pregnancies a Foley catheter constituted the preferable method of labor preinduction. Given its superior feasibility, reduced cost, and enhanced safety profile, this mechanistic approach should be advocated as the preferred intervention for individuals presenting with a favorable cervical status and risk factors for PPH.

The primary strength of the current investigation lies in its exhaustive assessment of factors associated with postpartum hemorrhage within a tertiary care institution. This approach facilitated the inclusion of multiple pregnancy pathologies and ensured the availability of advanced medical care. Furthermore, a comparative analysis was conducted on two highly accessible methods of EBL, suggesting the potential for replication of the study’s protocol in settings with limited resources. Nevertheless, it is imperative to address certain limitations. Firstly, due to a relatively small cohort and, as a result, the number of PPH cases, the study had limited power to detect differences in uncommon outcomes as well as to precisely estimate the effect of less frequent predictors (e.g., 3/4 degree perineal rupture, stillbirth, shoulder dystocia). Secondly, the limited number of PPH events relative to the number of variables included in the regression analysis raises the possibility of model overfitting, and thus the identified associations should be interpreted with caution. It should also be acknowledged that certain observations, such as the effect of dinoprostone on the risk of PPH, could be affected by confounding variables like the indication for labor induction. Regarding study design, this was a single-center study conducted in a tertiary care institution, which may limit the generalizability of the findings to other obstetric settings. Finally, the studied cohort was not representative of the general obstetric population, as patients in multiple pregnancies or those undergoing CS were excluded from the analysis.

## 5. Conclusions

The conducted analysis revealed a substantial discrepancy between clinician-based visual estimation and formula-derived calculations of blood loss, indicating a tendency for the former to underestimate and the latter to overestimate actual values. As a consequence, there is a clear need to improve methods of blood loss assessment, as neither sEBL nor fEBL alone provides a sufficiently accurate tool for clinical decision-making. The combined evaluation of lost blood volume and awareness of predisposing factors are essential elements for the successful prediction, early identification, and clinical management of excessive maternal bleeding following vaginal delivery.

## Figures and Tables

**Table 1:** Maternal characteristics of the study population.

	PPH (n = 29)	Control Group (n = 456)	*p* Value
Age (years) ^a^	31	31	0.85
	[28–35]	[28–34]	
<30 ^b^	12 (41.4%)	159 (34.9%)	0.67
30–34 ^b^	9 (31%)	188 (41.2%)	
35–39 ^b^	7 (24.1%)	86 (18.9%)	
≥40 ^b^	1 (3.4%)	23 (5%)	
Gravidity ^a^	1	2	<0.05
	[1, 2]	[1–3]	
Parity ^a^	1	2	<0.05
	[1, 2]	[1, 2]	
1 ^b^	20 (69%)	217 (47.6%)	0.07
2 ^b^	8 (27.6%)	176 (38.6%)	
≥3 ^b^	1 (3.4%)	63 (13.8%)	
Pre-pregnancy weight (kg) ^a^	64	63	0.62
	[57–70]	[57–71]	
Gestational weight gain (kg) ^a^	14	13	0.06
	[10–18]	[10–17]	
<10 ^b^	7 (24.1%)	108 (23.7%)	0.58
10–14.99 ^b^	11 (37.9%)	178 (39.1%)	
15–19.99 ^b^	6 (20.7%)	124 (27.3%)	
≥20 ^b^	5 (17.2%)	45 (9.9%)	
Height (m) ^a^	1.7	1.68	<0.05
	[1.64–1.75]	[1.64–1.72]	
Pre-pregnancy BMI (kg/m^2^) ^a^	22.28	22.31	0.88
	[20.7–24.6]	[20.4–25]	
<25 ^b^	22 (75.9%)	342 (75%)	0.37
≥25–29.9 ^b^	3 (10.3%)	78 (17.1%)	
≥30 ^b^	4 (13.8%)	36 (7.9%)	
GDM ^b^	8 (27.6%)	72 (15.8%)	0.16
PGDM ^b^	0 (0%)	6 (1.3%)	0.99
PIH ^b^	0 (0%)	17 (3.7%)	0.61
Chronic hypertension ^b^	0 (0%)	16 (3.5%)	0.61
Kidney/liver transplant recipient ^b^	0 (0%)	2 (0.4%)	0.99

Data are expressed as median and [IQR] or as frequency (%). ^a^—continuous variable; ^b^—categorical variable. BMI—body mass index; GDM—gestational diabetes mellitus; PGDM—pregestational diabetes mellitus; PIH—pregnancy induced hypertension; PPH—post-partum hemorrhage; IQR—interquartile range.

**Table 2:** Pregnancy and delivery-related outcomes in the study population.

	PPH (n = 29)	Control Group (n = 456)	*p* Value
Gestational age (weeks) ^a^	40	39	0.26
	[39, 40]	[39, 40]	
Pre-term delivery < 37 weeks ^b^	2 (6.9%)	19 (4.2%)	0.36
Delivery ^b^:			
Spontaneous	15 (51.7%)	295 (64.7%)	0.23
Induced	14 (48.3%)	161 (35.3%)	
Methods of VD preinduction/induction ^b,^*:			
Foley catheter	6 (42.9%)	103 (64%)	0.20
Dinoprostone insert	9 (64.3%)	47 (29.2%)	<0.05
Amniocentesis	0 (0%)	25 (15.5%)	0.22
Oxytocin administration	5 (35.7%)	78 (48.4%)	0.41
PROM ^b^	8 (27.6%)	109 (23.9%)	0.82
VBAC ^b^	0 (0%)	19 (4.2%)	0.62
Preeclampsia ^b^	1 (3.4%)	6 (1.3%)	0.35
I stage of delivery time (min.) ^a^	270	270	0.91
	[170–375]	[173.7–420]	
II stage of delivery time (min.) ^a^	61	33.5	<0.05
	[25–99]	[16–70]	
<120 ^b^	23 (79.3%)	405 (88.8%)	<0.05
120–179 ^b^	3 (10.3%)	43 (9.4%)	
≥180 ^b^	3 (10.3%)	8 (1.8%)	
III stage of delivery time (min.) ^a^	13	7	<0.001
	[8–30]	[5–10]	
<30 ^b^	21 (72.4%)	439 (96.3%)	<0.001
≥30 ^b^	8 (27.6%)	17 (3.7%)	
Total time of delivery (min.) ^a^	385	319	0.34
	[217–556]	[211.5–486.2]	
Night shift ^b^	13 (44.8%)	145 (31.8%)	0.21
Epidural anesthesia ^b^	25 (86.2%)	324 (71.1%)	0.09
Vacuum delivery ^b^	4 (13.8%)	35 (7.7%)	0.28
Episiotomy ^b^	11 (37.9%)	151 (33.1%)	0.74
Shoulder dystocia ^b^	0 (0%)	1 (0.2%)	0.99
Perineal rupture degree ^b^:			
1	10 (34.5%)	109 (23.9%)	0.29
2	4 (13.8%)	45 (9.9%)	0.52
3/4	2 (6.9%)	0 (0%)	<0.01
Oxytocin prophylaxis ^b^	25 (86.2%)	421 (92.3%)	0.28
Uterine atony ^b^	12 (41.4%)	7 (1.5%)	<0.001
Carbetocin ^b^	16 (55.2%)	50 (11%)	<0.001
Methergine ^b^	8 (27.6%)	4 (0.9%)	<0.001
Misoprostol ^b^	12 (41.4%)	7 (1.5%)	<0.001
Tranexamic acid ^b^	16 (55.2%)	33 (7.2%)	<0.001
Retained placenta ^b^	8 (27.6%)	10 (2.2%)	<0.001
RPOC ^b^	18 (62.1%)	34 (7.5%)	<0.001
Curettage ^b^	28 (96.6%)	36 (7.9%)	<0.001
Crede maneuver ^b^	8 (27.6%)	10 (2.2%)	<0.001
Manual removal of the placenta ^b^	8 (27.6%)	6 (1.3%)	<0.001
Blood transfusion ^b^	6 (20.7%)	4 (0.9%)	<0.001

Data are expressed as median and [IQR] or as frequency (%). ^a^—continuous variable; ^b^—categorical variable. *—calculated as percentage of women qualified for labor induction. PROM—premature rupture of membranes; PPH—post-partum hemorrhage; VBAC—vaginal birth after Cesarean section; RPOC—retained products of conception; VD—vaginal delivery; IQR—interquartile range.

**Table 3:** Fetal and neonatal characteristics of the study population.

	PPH (n = 29)	Control Group (n = 456)	*p* Value
FGR ^b^	0 (0%)	17 (3.7%)	0.61
SGA ^b^	0 (0%)	8 (1.8%)	0.99
FBW (g) ^a^	3420	3395	0.55
	[3120–3800]	[3120–3660]	
<3000 ^b^	6 (20.7%)	81 (17.8%)	<0.05
3000–3499 ^b^	12 (41.4%)	194 (42.5%)	
3500–3999 ^b^	6 (20.7%)	159 (34.9%)	
≥4000 ^b^	5 (17.2%)	22 (4.8%)	
Fetal macrosomia ≥ 4000 g ^b^	5 (17.2%)	22 (4.8%)	<0.05
Fetal sex ^b^			
Male	13 (44.8%)	230 (50.4%)	0.69
Female	16 (55.2%)	226 (49.6%)	
Stillbirth ^b^	2 (6.9%)	1 (0.2%)	<0.05
1st minute Apgar ^a^	10	10	0.77
	[10, 10]	[10, 10]	
5th minute Apgar ^a^	10	10	0.37
	[10, 10]	[10, 10]	
10th minute Apgar ^a^	10	10	0.11
	[10, 10]	[10, 10]	
Umbilical artery pH ^a^	7.26	7.24	0.11
	[7.23–7.34]	[7.2–7.3]	

Data are expressed as median and [IQR] or as frequency (%). ^a^—continuous variable; ^b^—categorical variable. FBW—fetal birthweight; FGR—fetal growth-restriction; SGA—small-for-gestational age; PPH—post-partum hemorrhage; IQR—interquartile range.

**Table 4:** Estimated blood loss according to the type of method applied.

Group	sEBL (mL)	fEBL (mL)	*p* Value	Effect Size
PPH (n = 29)	800	1439.6	<0.001	0.61
	[600–1000]	[1114.3–1673.6]		
Control group (n = 456)	250	621.8	<0.001	0.52
	[200–300]	[330.1–920]		

Data are expressed as median and [IQR]. PPH—post-partum hemorrhage; sEBL—visually estimated blood loss; fEBL—formula estimated blood loss; IQR—interquartile range.

**Table 5:** Univariate analysis of factors contributing to early post-partum hemorrhage following vaginal delivery.

Factor	Estimate	OR	95% CI	*p* Value
Age (years)				
30–34	−0.46	0.63	0.26–1.54	0.32
35–39	0.08	1.08	0.41–2.84	0.88
≥40	−0.55	0.58	0.07–4.64	0.60
(reference group: age < 30 years)				
Pre-term delivery < 37 weeks	0.53	1.7	0.38–7.7	0.49
(reference group: time of delivery ≥ 37 weeks)				
Parity				
2	−0.71	0.49	0.21–1.15	0.10
≥3	−1.76	0.17	0.02–1.31	0.09
(reference group: parity 1)				
Gestational weight gain (kg)				
10–14.99	−0.05	0.95	0.36–2.53	0.92
15–19.99	−0.29	0.75	0.24–2.29	0.61
≥20	0.54	1.71	0.52–5.69	0.38
(reference group: gestational weight gain < 10 kg)				
Pre-pregnancy BMI (kg/m^2^)				
25–29.9	−0.51	0.59	0.17–2.05	0.41
≥30	0.55	1.73	0.56–5.29	0.34
(reference group: pre-pregnancy BMI < 25 kg/m^2^)				
Delivery				
Spontaneous	−0.54	0.58	0.27–1.24	0.16
Induced	0.54	1.71	0.8–3.63	0.16
Method of VD preinduction/induction:				
Foley catheter	−0.11	0.89	0.35–2.25	0.81
Dinoprostone	1.34	3.83	1.65–8.87	<0.01
Amniocentesis	−0.64	0.53	0.07–4	0.53
Oxytocin administration	−0.02	0.98	0.42–2.26	0.95
PROM	0.19	1.21	0.52–2.82	0.65
Preeclampsia	0.99	2.68	0.31–23	0.37
GDM	0.71	2.03	0.87–4.76	0.10
II stage of delivery time (min.)				
120–179	0.21	1.23	0.35–4.26	0.75
≥180	1.89	6.6	1.64–26.56	<0.01
(reference group: II stage of delivery < 120 min.)				
III stage of delivery time (min.)				
≥30	2.29	9.84	3.81–25.4	<0.001
(reference group: III stage of delivery < 30 min.)				
Night shift	0.56	1.74	0.82–3.72	0.15
Epidural anesthesia	0.94	2.55	0.87–7.46	0.09
Vacuum delivery	0.66	1.92	0.63–5.84	0.25
Episiotomy	0.21	1.23	0.57–2.68	0.59
Perineal rupture degree:				
1	0.52	1.68	0.76–3.71	0.20
2	0.38	1.46	0.49–4.39	0.49
Erythrocytes diff.—≥0.7 mln/dL	2.48	11.9	4.73–30	<0.001
(reference group: erythrocytes diff. < 0.7 mln/dL)				
Hgb diff.—≥2 g/dL	2.55	12.9	4.8–34.4	<0.001
(reference group: hgb diff. < 2 g/dL)				
Hct diff.—≥7 %	2.51	12.3	5.28–28.8	<0.001
(reference group: hct diff. <7%)				
FBW (g)				
3000–3499	−0.18	0.83	0.30–2.3	0.73
3500–3999	−0.67	0.51	0.16–1.63	0.26
≥4000	1.12	3.07	0.86–11	<0.05
(reference group: FBW < 3000 g)				
Stillbirth	3.52	3.37	0.3–38.3	<0.01

BMI—body mass index; PROM—premature rupture of membranes; GDM—gestational diabetes mellitus; RPOC—retained products of conception; FBW—fetal birth-weight; VD—vaginal delivery; erythrocytes diff.—difference between pre- and post-delivery concentrations of erythrocytes; Hgb diff.—difference between pre- and post-delivery hemoglobin levels; Hct diff.—difference between pre- and post-delivery hematocrit; OR—odds ratio; CI—confidence interval.

**Table 6:** Factors associated with the early post-partum hemorrhage following vaginal delivery: results from the multivariate analysis (AUC 0.779; 95%CI: 0.685–0.872).

Factor	Estimate	OR	95% CI	*p* Value
Dinoprostone insert	1.47	4.33	1.63–11.48	<0.01
III stage of delivery time ≥ 30 min.	2.45	11.62	4.18–32.33	<0.001
FBW 3000–3499 g	0.06	1.06	0.34–3.29	0.92
FBW 3500–3999 g	−0.65	0.52	0.14–1.9	0.32
FBW ≥ 4000 g	1.85	6.37	1.54–26.3	<0.05

FBW—fetal birth-weight; OR—odds ratio; CI—confidence interval.

## Data Availability

The datasets used and/or analyzed during the current study are available from the corresponding author on reasonable request.

## Supplementary Materials

The following supporting information can be downloaded at: https://www.mdpi.com/article/10.3390/jcm15083000/s1, Figure S1. Heatmaps of correlations between selected maternal-fetal parameters and methods of blood loss estimation (A—study + control group, B—control group, C—study group). BMI—body mass index; EBL—estimated blood loss; Hgb—hemoglobin; Hct—hematocrit. Heatmaps use colors and sizes to represent data values [smaller points represent weak correlations, while larger points indicate higher correlation strength; warmer colors (e.g., red, orange) reflect negative correlations, whereas cooler colors (blue shades) indicate positive correlations]. * *p* < 0.05; ** *p* < 0.01; *** *p* < 0.001; Table S1. Selected maternal laboratory data in the studied population.

## References

[1] MBRACE-UK (2023). Saving Lives, Improving Mothers’ Care Core Report: Lessons Learned to Inform Maternity Care from the UK and Ireland Confidential Enquiries into Maternal Deaths and Morbidity 2019–2021.

[2] Ueda A., Nakakita B., Chigusa Y., Mogami H., Ohtera S., Kato G., Mandai M., Kondoh E. (2022). Impact of efforts to prevent maternal deaths due to obstetric hemorrhage on trends in epidemiology and management of severe postpartum hemorrhage in Japan: A nationwide retrospective study. BMC Pregnancy Childbirth.

[3] Bienstock J.L., Eke A.C., Hueppchen N.A. (2021). Postpartum Hemorrhage. N. Engl. J. Med..

[4] World Health Organization (2012). WHO Recommendations for the Prevention and Treatment of Postpartum Haemorrhage.

[5] American College of Obstetricians and Gynecologists (2017). Practice Bulletin No. 183: Postpartum hemorrhage. Obstet. Gynecol..

[6] Sentilhes L., Goffinet F., Vayssière C., Deneux-Tharaux C. (2017). Comparison of postpartum haemorrhage guidelines: Discrepancies underline our lack of knowledge. BJOG.

[7] Sheldon W.R., Blum J., Vogel J.P., Souza J.P., Gülmezoglu A.M., Winikoff B. (2014). Postpartum haemorrhage management, risks, and maternal outcomes: Findings from the World Health Organization Multicountry Survey on Maternal and Newborn Health. BJOG.

[8] Bell S.F., Watkins A., John M., Macgillivray E., Kitchen T.L., James D., Scarr C., Bailey C.M., Kelly K.P., James K. (2020). Incidence of postpartum haemorrhage defined by quantitative blood loss measurement: A national cohort. BMC Pregnancy Childbirth.

[9] Yunas I., Islam M.A., Price M.J., Melo P., Aswat A., Alam S.S., Kundu S., Oladapo O.T., Zamora J., Gallos I.D. (2025). Prevalence of postpartum haemorrhage: A systematic review and meta-analysis. Lancet Obstet. Gynaecol. Women’s Health.

[10] Evensen A., Anderson J.M., Fontaine P. (2017). Postpartum Hemorrhage: Prevention and Treatment. Am. Fam. Physician.

[11] Grönvall M., Tikkanen M., Tallberg E., Paavonen J., Stefanovic V. (2013). Use of Bakri balloon tamponade in the treatment of postpartum hemorrhage: A series of 50 cases from a tertiary teaching hospital. Acta Obstet. Gynecol. Scand..

[12] Legendre G., Richard M., Brun S., Chancerel M., Matuszewski S., Sentilhes L. (2016). Evaluation by obstetric care providers of simulated postpartum blood loss using a collector bag: A French prospective study. J. Matern. Fetal Neonatal Med..

[13] Gerdessen L., Meybohm P., Choorapoikayil S., Herrmann E., Taeuber I., Neef V., Raimann F.J., Zacharowski K., Piekarski F. (2021). Comparison of common perioperative blood loss estimation techniques: A systematic review and meta-analysis. J. Clin. Monit. Comput..

[14] Perinatolog.com. Perinatology EBL Calculator; Calculated Blood Loss Calculator. https://www.perinatology.com/.

[15] Begum F., Nieto-Calvache A.J., Schlembach D., Hofmyer J., Palacios-Jaraquemada J., Bhardwaj A., Suarez M.A., Burgos-Luna J.M., Beyeza-Kashesya J., Ubom A.E. (2025). FIGO recommendations on objective measurement of blood loss after birth for early detection of postpartum hemorrhage. Int. J. Gynaecol. Obstet..

[16] Stafford I., Dildy G.A., Clark S.L., Belfort M.A. (2008). Visually estimated and calculated blood loss in vaginal and cesarean delivery. Am. J. Obstet. Gynecol..

[17] Feduniw S., Warzecha D., Szymusik I., Wielgos M. (2020). Epidemiology, prevention and management of early postpartum hemorrhage—A systematic review. Ginekol. Pol..

[18] Giouleka S., Tsakiridis I., Kalogiannidis I., Mamopoulos A., Tentas I., Athanasiadis A., Dagklis T. (2022). Postpartum Hemorrhage: A comprehensive review of guidelines. Obstet. Gynecol. Surv..

[19] Yunas I., Islam M.A., Sindhu K.N., Devall A.J., Podesek M., Alam S.S., Kundu S., Mammoliti K.M., Aswat A., Price M.J. (2025). Causes of and risk factors for postpartum haemorrhage: A systematic review and meta-analysis. Lancet.

[20] Patek K., Friedman P. (2023). Postpartum hemorrhage-epidemiology, risk factors, and causes. Clin. Obstet. Gynecol..

[21] Huang C.R., Xue B., Gao Y., Yue S.W., Redding S.R., Wang R., Ouyang Y.Q. (2023). Incidence and risk factors for postpartum hemorrhage after vaginal delivery: A systematic review and meta-analysis. J. Obstet. Gynaecol. Res..

[22] Kwiatkowski S., Huras H., Fuchs T., Sokołowska M., Oszukowski P., Stojko R., Czajkowski K., Kamiński P., Sieroszewski P., Drews K. (2022). Rekomendacje Polskiego Towarzystwa Ginekologów i Położników. Postępowania w przypadku wystąpienia krwotoków okołoporodowych. Ginekol. Perinatol. Prakt..

[23] Borovac-Pinheiro A., Pacagnella R.C., Cecatti J.G., Miller S., El Ayadi A.M., Souza J.P., Durocher J., Blumenthal P.D., Winikoff B. (2018). Postpartum hemorrhage: New insights for definition and diagnosis. Am. J. Obstet. Gynecol..

[24] Bomba-Opoń D., Drews K., Huras H., Laudański P., Paszkowski T., Wielgoś M. (2017). Polish Gynecological Society Recommendations for Labor Induction. Ginekol. Pol..

[25] Wielgos M., Bomba-Opoń D., Breborowicz G.H., Czajkowski K., Debski R., Leszczynska-Gorzelak B., Oszukowski P., Radowicki S., Zimmer M. (2018). Recommendations of the Polish Society of Gynecologists and Obstetricians regarding caesarean sections. Ginekol. Pol..

[26] Gordijn S.J., Beune I.M., Thilaganathan B., Papageorghiou A., Baschat A.A., Baker P.N., Silver R.M., Wynia K., Ganzevoort W. (2016). Consensus definition of fetal growth restriction: A Delphi procedure. Ultrasound Obstet. Gynecol..

[27] Carroli G., Cuesta C., Abalos E., Gulmezoglu A.M. (2008). Epidemiology of postpartum haemorrhage: A systematic review. Best Pract. Res. Clin. Obstet. Gynaecol..

[28] Al Kadri H.M., Al Anazi B.K., Tamim H.M. (2011). Visual estimation versus gravimetric measurement of postpartum blood loss: A prospective cohort study. Arch. Gynecol. Obstet..

[29] Dildy G.A., Paine A.R., George N.C., Velasco C. (2004). Estimating blood loss: Can teaching significantly improve visual estimation?. Obstet. Gynecol..

[30] Al-Kadri H.M., Dahlawi H., Al Airan M., Elsherif E., Tawfeeq N., Mokhele Y., Brown D., Tamim H.M. (2014). Effect of education and clinical assessment on the accuracy of post partum blood loss estimation. BMC Pregnancy Childbirth.

[31] Natrella M., Di Naro E., Loverro M., Benshalom-Tirosh N., Trojano G., Tirosh D., Besser L., Loverro M.T., Mastrolia S.A. (2018). The more you lose the more you miss: Accuracy of postpartum blood loss visual estimation. A systematic review of the literature. J. Matern. Fetal Neonatal Med..

[32] Rubenstein A.F., Zamudio S., Douglas C., Sledge S., Thurer R.L. (2021). Automated quantification of blood loss versus visual estimation in 274 vaginal deliveries. Am. J. Perinatol..

[33] Włodarczyk Z., Śliwka A., Maciocha H., Paruszewski S., Wyszyńska J., Kłopecka M., Afrykańska G., Śliwińska M., Ludwin A., Stanirowski P.J. (2025). The role of accurate estimations of blood loss and identification of risk factors in the management of early postpartum hemorrhage in women undergoing a cesarean section. J. Clin. Med..

[34] Kreutziger J., Puchner P., Schmid S., Mayer W., Prossliner H., Lederer W. (2021). Accuracy of training blood volume quantification using a visual estimation tool. World J. Emerg. Med..

[35] Madar H., Sentilhes L., Goffinet F., Bonnet M.P., Rozenberg P., Deneux-Tharaux C. (2023). Comparison of quantitative and calculated postpartum blood loss after vaginal delivery. Am. J. Obstet. Gynecol. MFM.

[36] Gari A., Hussein K., Daghestani M., Aljuhani S., Bukhari M., Alqahtani A., Almarwani M. (2022). Estimating blood loss during cesarean delivery: A comparison of methods. J. Taibah Univ. Med. Sci..

[37] Sel G., Arikan I.I., Harma M., Harma M.I. (2021). A new and feasible uterine compression suture technique in uterine atony to save mothers from postpartum hemorrhage. Niger. J. Clin. Pract..

[38] Said Ali A., Faraag E., Mohammed M., Elmarghany Z., Helaly M., Gadallah A., Taymour M.A., Ahmad Y., Ibrahim Eissa A., Ibrahim Ogila A. (2021). The safety and effectiveness of Bakri balloon in the management of postpartum hemorrhage: A systematic review. J Matern. Fetal Neonatal Med..

[39] Liu C.N., Yu F.B., Xu Y.Z., Li J.S., Guan Z.H., Sun M.N., Liu C.A., He F., Chen D.J. (2021). Prevalence and risk factors of severe postpartum hemorrhage: A retrospective cohort study. BMC Pregnancy Childbirth.

[40] Gallos I.D., Papadopoulou A., Man R., Athanasopoulos N., Tobias A., Price M.J., Williams M.J., Diaz V., Pasquale J., Chamillard M. (2018). Uterotonic agents for preventing postpartum haemorrhage: A network meta-analysis. Cochrane Database Syst. Rev..

[41] de Vries P.L.M., Veenstra E., Baud D., Legardeur H., Kallianidis A.F., van den Akker T. (2025). Time to redefine prolonged third stage of labor? A systematic review and meta-analysis of the length of the third stage of labor and adverse maternal outcome after vaginal birth. Am. J. Obstet. Gynecol..

[42] Magann E.F., Evans S., Chauhan S.P., Lanneau G., Fisk A.D., Morrison J.C. (2005). The length of the third stage of labor and the risk of postpartum hemorrhage. Obstet. Gynecol..

[43] Voillequin S., Quibel T., Rozenberg P., Rousseau A. (2025). Duration of the second and third stages of labor and risk of postpartum hemorrhage: A cohort study stratified by parity. BMC Pregnancy Childbirth.

[44] Perlman N.C., Carusi D.A. (2019). Retained placenta after vaginal delivery: Risk factors and management. Int. J. Womens Health.

[45] Patwardhan M., Hernandez-Andrade E., Ahn H., Korzeniewski S.J., Schwartz A., Hassan S.S., Romero R. (2015). Dynamic changes in the myometrium during the third stage of labor, evaluated using two-dimensional ultrasound, in women with normal and abnormal third stage of labor and in women with obstetric complications. Gynecol. Obstet. Investig..

[46] Beta J., Khan N., Khalil A., Fiolna M., Ramadan G., Akolekar R. (2019). Maternal and neonatal complications of fetal macrosomia: Systematic review and meta-analysis. Ultrasound Obstet. Gynecol..

[47] Quezada-Robles A., Quispe-Sarmiento F., Bendezu-Quispe G., Vargas-Fernández R. (2023). Fetal macrosomia and postpartum hemorrhage in Latin American and Caribbean region: Systematic review and meta-analysis. Rev. Bras. Ginecol. Obstet..

[48] Di Tommaso M., Pellegrini R., Ammar O., Lecis S., Huri M., Facchinetti F. (2025). Safety of the use of dinoprostone gel and vaginal insert for induction of labor: A multicenter retrospective cohort study. Int. J. Gynaecol. Obstet..

[49] Thomas J., Fairclough A., Kavanagh J., Kelly A.J. (2014). Vaginal prostaglandin (PGE2 and PGF2a) for induction of labour at term. Cochrane Database Syst. Rev..

