# Supplementary material for: Accuracy of Blood Loss Estimation and Identification of Factors Contributing to Early Postpartum Hemorrhage Following Vaginal Delivery
Source: J Clin Med. 2026 Apr 15;15(8):3000. doi: 10.3390/jcm15083000 (PMC13116601; doi:10.3390/jcm15083000)
Supplement: Supplementary file 1 [file jcm-15-03000-s001.zip › Supplementary Figure S1 .pdf]

Supplementary Figure S1. Heatmaps of correlations between selected maternal-fetal parameters and methods of blood loss estimation (A – study + control group, B – control group, C – study group). BMI – body mass index; EBL – estimated blood loss; Hgb – hemoglobin; Hct - hematocrit. Heatmaps use colors and sizes to represent data values [smaller points represent weak correlations, while larger points indicate higher correlation strength; warmer colors (e.g., red, orange) reflect negative correlations, whereas cooler colors (blue shades) indicate positive correlations]. * - p<.05; ** - p<.01; *** - p<.001

A

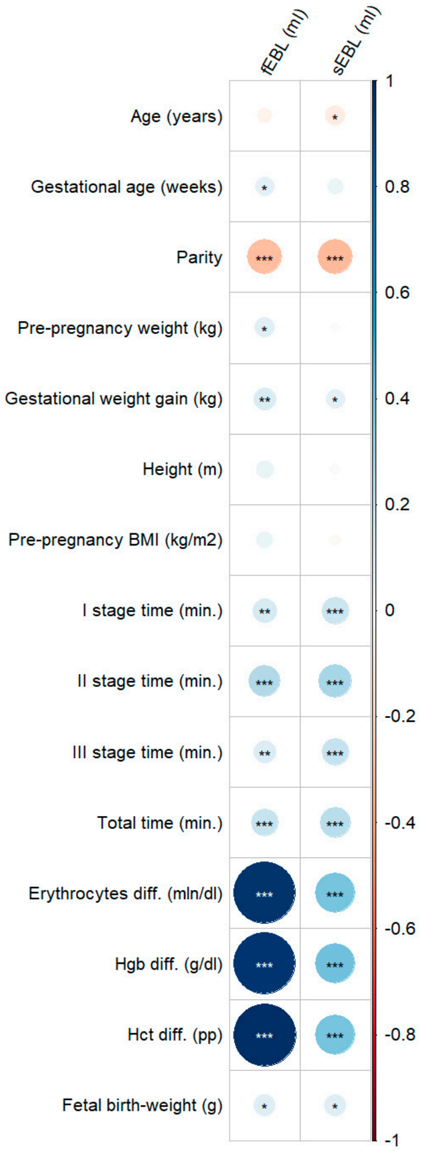

B

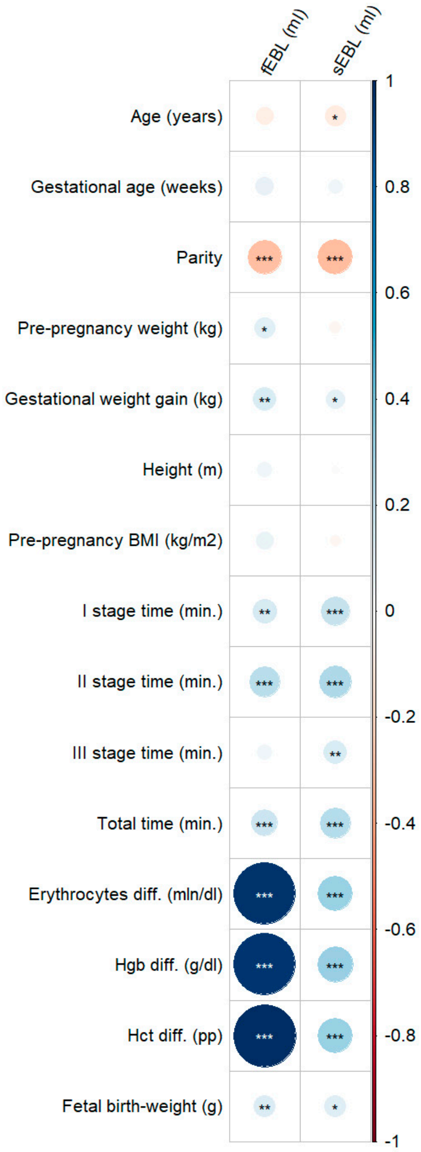

fEBL (ml)
sEBL (ml)
Age (years)
Gestational age (weeks)
Parity
Pre-pregnancy weight (kg)
Gestational weight gain (kg)
Height (m)
Pre-pregnancy BMI (kg/m2)
I stage time (min.)
II stage time (min.)
III stage time (min.)
Total time (min.)
Erythrocytes diff. (mln/dl)
Hgb diff. (g/dl)
Hct diff. (pp)
Fetal birth-weight (g)
1
0.8
0.6
0.4
0.2
0
-0.2
-0.4
-0.6
-0.8
-1

C

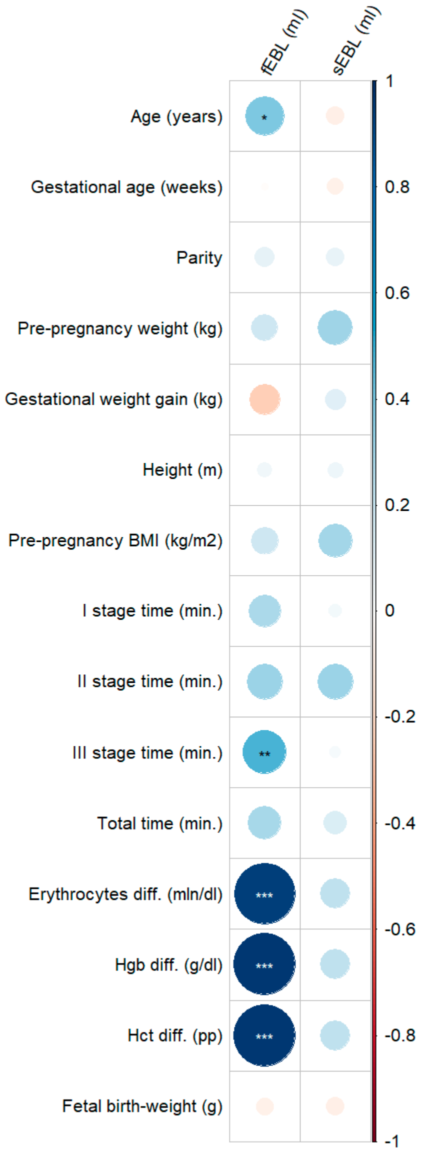

fEBL (ml)
sEBL (ml)
Age (years)
Gestational age (weeks)
Parity
Pre-pregnancy weight (kg)
Gestational weight gain (kg)
Height (m)
Pre-pregnancy BMI (kg/m2)
I stage time (min.)
II stage time (min.)
III stage time (min.)
Total time (min.)
Erythrocytes diff. (mln/dl)
Hgb diff. (g/dl)
Hct diff. (pp)
Fetal birth-weight (g)
*
**
***
***
***
1
0.8
0.6
0.4
0.2
0
-0.2
-0.4
-0.6
-0.8
-1
