# Supplementary material for: Accuracy of Blood Loss Estimation and Identification of Factors Contributing to Early Postpartum Hemorrhage Following Vaginal Delivery
Source: J Clin Med. 2026 Apr 15;15(8):3000. doi: 10.3390/jcm15083000 (PMC13116601; doi:10.3390/jcm15083000)
Supplement: Supplementary file 1 [file jcm-15-03000-s001.zip › Supplementary Table S1.pdf]

Supplementary Table S1. Selected maternal laboratory data in the studied population.

	PPH (n=29)	Control group (n=456)	p Value
Erythrocytes before VD (mln/dl)[a]	4.19 [3.89-4.45]	4.18 [3.95-4.43]	.92
Erythrocytes after VD (mln/dl)[a]	3.08±0.48 (1.82-3.96)	3.67±0.39 (2.4-4.75)	<.001
Erythrocytes diff. (mln/dl)[a]	0.97 [0.75-1.34]	0.48 [0.27-0.69]	<.001
<0.7[b]	6 (20.7%)	345 (75.7%)	<.001
≥0.7[b]	23 (79.3%)	111 (24.3%)	
Hgb concentration before VD (g/dl)[a]	12.5 [12.4-13.5]	12.5 [11.9-13.2]	.19
Hgb concentration after VD (g/dl)[a]	9.54±1.38 (6.2-12.9)	11.1±1.14 (7.4-14.5)	<.001
Hgb diff. (g/dl)[a]	2.8 [2.2-3.9]	1.3 [0.8-2]	<.001
<2[b]	5 (17.2%)	332 (72.8%)	<.001
≥2[b]	24 (82.8%)	124 (27.2%)	
Hct before VD (%)[a]	37.1 [35.8-38.9]	36.6 [35.1-28.6]	.41
Hct after VD (%)[a]	27.5±3.95 (17.5-36.8)	32.5±3.16 (23.9-41.1]	<.001
Hct diff. (%)[a]	8.7 [6.5-11.4]	3.9 [2.1-6]	<.001
<7[b]	8 (27.6%)	376 (82.5%)	<.001
≥7[b]	21 (72.4%)	80 (17.5%)	

Data are expressed as mean ± SD/ (range); median and [IQR] or as frequency (%).
(a) – continuous variable; (b) – categorical variable
Hgb - hemoglobin concentration; *Hct* – hematocrit; *erythrocytes diff.* - difference between pre- and post-delivery concentrations of erythrocytes; *Hgb diff.* - difference between pre- and post-delivery hemoglobin levels; *Hct diff.* - difference between pre- and post-delivery hematocrit; *PPH* – post-partum hemorrhage; *SD* - standard deviation; *VD* – vaginal delivery; *IQR* - interquartile range
